# Supplementary material for: Long Non-Coding RNAs Expressed in the Peanut Allergy for Understanding the Pathophysiology of Peanut Allergy Rat Model
Source: Foods. 2022 Nov 22;11(23):3760. doi: 10.3390/foods11233760 (PMC9740276; doi:10.3390/foods11233760)
Supplement: Supplementary file 1 [file foods-11-03760-s001.zip › foods-1995380-supplementary.pdf]

Supplementary Table S1 Primer Sequences for qRT-PCR

Gene	Primer sequences (5'-3')	
	Forward	Reverse
ENSRNOT00000093217	TTTCATCGCGGATCCAGTTG	TTGTCGTCCGCATTGTCATC
ENSRNOT00000087227	ACATCGTTTCTTGGCAACCG	AAAAGCTGAACCACGCTTCC
ENSRNOT00000057756	AAGGGGTTTGCAGCATCAAG	ATGGCGTTGAAGTTGAGCTG
ENSRNOT00000090335	GCAGCCTTGAAACTGTCTTCTG	TCAGCCATTGTGGAAGAAGC
ENSRNOT00000085965	TGACTTGCAGCATGATCACC	TTGAAAGCGCCTGTGTGAAG
ENSRNOT00000081904	AGGAATTTCTCTGCCGACCG	GTAACCCTGGTTCTGACCCG
ENSRNOT00000074532	TTGGCAAGAAGAGGCAAACC	ACGTCTTGCCAAACAGACAG
ENSRNOT00000075924	TGCCGACTTGCTCGAATTTG	TTACAGGCCAGAAGGAAGACAC
β actin	AAGTGTGACGTTGACATCCGTAAAG	CAGCTCAGTAACAGTCCGCCTAGA

Supplementary Table S2 Top 20 upregulated lncRNAs between control and PA group

ID	Log2FoldChange	P value	regulated
LNC_000973	15.000487	5.69E-29	up
LNC_001137	11.8774465	0.01043194	up
LNC_000572	11.7236541	0.01159083	up
LNC_001066	10.0829908	2.90E-06	up
LNC_000731	8.51157772	0.00037136	up
LNC_000784	8.0774741	0.00056011	up
ENSRNOT00000085965	4.52387407	0.00970828	up
LNC_000429	4.44022057	0.00405215	up
LNC_000830	4.39794969	0.00618489	up
LNC_000428	3.736681	0.03047347	up
LNC_000735	3.46288526	0.0037871	up
LNC_000741	3.44286832	0.02464223	up
LNC_001082	2.9732219	9.49E-05	up
LNC_000441	2.96960037	2.54E-06	up
LNC_000362	2.91700069	1.01E-08	up
LNC_000785	2.91188724	1.28E-05	up
LNC_000863	2.85661473	1.18E-09	up
LNC_001041	2.76724782	0.02755445	up
LNC_001274	2.72168895	7.85E-07	up

Supplementary Table S3 Top 20 downregulated lncRNAs between control and PA group

ID	Log2FoldChange	P value	regulated
ENSRNOT00000075902	-10.011099	1.13E-13	down
ENSRNOT00000060194	-9.8634919	1.96E-13	down
LNC_000961	-9.3909015	0.04955173	down
LNC_000516	-8.0773944	2.08E-06	down
ENSRNOT00000076640	-7.9512526	9.51E-08	down
ENSRNOT00000077291	-7.2655089	1.99E-06	down
LNC_000517	-4.4162131	0.00020716	down
ENSRNOT00000091124	-4.2606053	0.00106396	down
ENSRNOT00000093265	-3.1570083	0.00299561	down
ENSRNOT00000088578	-3.0974466	0.00788707	down
ENSRNOT00000092617	-3.0157254	0.0002969	down
LNC_000564	-3.0142988	0.00153503	down
ENSRNOT00000083231	-2.8627447	1.71E-06	down
LNC_000211	-2.5447015	0.00118136	down
LNC_000018	-2.5082382	6.16E-13	down
LNC_001061	-2.4994819	0.00182251	down
LNC_000537	-2.3225284	0.00241768	down
LNC_000997	-2.1908221	2.18E-07	down
LNC_000718	-2.0970148	0.00218875	down
LNC_000044	-2.0800396	0.00081224	down
